# Cell-free expression of RuBisCO for ATP production in the synthetic cells

**DOI:** 10.1093/synbio/ysad016

**Published:** 2023-12-20

**Authors:** Shugo Sugii, Katsumi Hagino, Ryo Mizuuchi, Norikazu Ichihashi

**Affiliations:** Department of Life Science, Graduate School of Arts and Science, The University of Tokyo, Meguro, Tokyo 153-8902, Japan; Department of Electrical Engineering and Bioscience, Faculty of Science and Engineering, Waseda University, Shinjuku, Tokyo 162-8480, Japan; JST FOREST, Kawaguchi, Saitama 332-0012, Japan; Department of Life Science, Graduate School of Arts and Science, The University of Tokyo, Meguro, Tokyo 153-8902, Japan; Komaba Institute for Science, The University of Tokyo, Meguro, Tokyo 153-8902, Japan; Universal Biology Institute, The University of Tokyo, Meguro, Tokyo 153-8902, Japan; College of Arts and Science, the University of Tokyo, Meguro, Tokyo 153-8902, Japan; Department of Medicine, the University of Tokyo, Bunkyo, Tokyo 113-8654, Japan

**Keywords:** RuBisCO, ATP production, CO_2_ fixation, artificial cell, *in vitro* synthetic biology

## Abstract

Recent advances in bottom-up synthetic biology have made it possible to reconstitute cellular systems from non-living components, yielding artificial cells with potential applications in industry, medicine and basic research. Although a variety of cellular functions and components have been reconstituted in previous studies, sustained biological energy production remains a challenge. ATP synthesis via ribulose-1,5-diphosphate carboxylase/oxygenase (RuBisCO), a central enzyme in biological CO_2_ fixation, holds potential as an energy production system, but its feasibility in a cell-free expression system has not yet been tested. In this study, we test RuBisCO expression and its activity-mediated ATP synthesis in a reconstituted *Escherichia coli*-based cell-free translation system. We then construct a system in which ATP is synthesized by RuBisCO activity in giant vesicles and used as energy for translation reactions. These results represent an advance toward independent energy production in artificial cells.

**Graphical Abstract** 
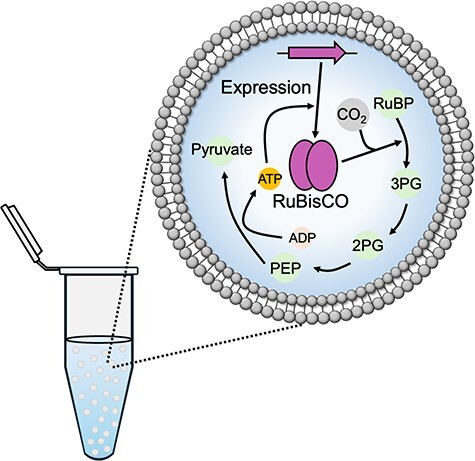

## 1. Introduction

Recent developments in bottom-up synthetic biology have led to the construction of artificial cells with a variety of capabilities to understand the design principles of biological systems and to develop new biotechnologies (1–15). To make these artificial cells more life-like, it is important to integrate functional modules such as gene expression, genome replication and metabolism (14). In particular, to sustain these various reactions, it is ideal to provide sufficient biological energy to the artificial cells in an autotrophic manner by reconstituting metabolic cascades such as photosynthesis and respiration (15–22). Moreover, the development of artificial cells has the potential to remodel metabolic systems that are different from the energy production systems of living organisms (23).

Ribulose-1,5-bisphosphate carboxylase/oxygenase (RuBisCO) is one of the central enzymes in biological CO_2_ fixation, catalyzing the reaction to convert CO_2_ and ribulose 1,5-bisphosphate (RuBP) to 3-phosphoglycerate (3PG) (24). Then, 3PG is converted to pyruvate by glycolytic enzymes, producing a single molecule of ATP (25). RuBisCO-mediated ATP synthesis has the potential to be used as an energy production method and can also be developed into an environmentally benign artificial cell that can consume CO_2_ in the atmosphere. However, the activity and ATP productivity of RuBisCO expressed in a cell-free translation system have not been verified yet.

In this study, we attempted to construct a system that fixes CO_2_ and synthesizes ATP using RuBisCO expressed in an *Escherichia coli*-based cell-free reconstituted translation system (PURE system) (26, 27). First, we detected the activity of RuBisCO derived from a photosynthetic bacterium *Rhodospirillum rubrum*. Next, we synthesized ATP by combining the cell-free expression of RuBisCO with glycolytic enzymes. Finally, we demonstrated that ATP synthesized by RuBisCO activity can be used as an energy source for cell-free expression systems in giant vesicles.

## 2. Matrials and methods

### 2.1 DNA preparation

The linear DNA-encoding RuBisCO under the T7 promoter used in this study was prepared as follows. First, the amino acid sequences of the RuBisCO genes of *Synechococcus elongatus* PCC6301 (*cbbL, cbbS*) and *R. rubrum* (*cbbM*) were obtained from the NCBI database. After optimizing the nucleotide sequences for *E. coli* translation, the genes and upstream T7 promoters were synthesized and inserted into the pEX vector using the artificial gene synthesis service of eurofins genomics. The PCR-amplified DNA fragments were prepared using primers 1 and 2 and these plasmids as templates. The PCR fragments were purified using the QIAquick PCR Purification Kit (QIAGEN), which was used for all DNA purification procedures in this study. The linear DNA fragment-encoding GFP used in Figures 3 and 4 was prepared by PCR using primers 3 and 4 with a plasmid-encoding GFP (pETG5tag) constructed in a previous study as a template (28). DNA concentration was calculated by measuring the absorbance (A_260_) using NanoDrop One (Thermo Fisher Scientific). All PCR reactions in this study were performed by using Prime STAR HS DNA Polymerase (Takara, Japan) according to the manufacturer’s instruction. The primer sequences and the plasmid sequences are shown in the Supplementary file, respectively.

### 2.2 NADH absorbance assay

The assay methods were based on a previous study (29). First, the linear DNA encoding each RuBisCO (5 nM) was incubated at 30°C for 4 h using a commercially available PURE system (PUREfrex 2.0, GeneFrontier) to express RuBisCOs. *CbbL* and *cbbS* were expressed independently and mixed at a ratio of 1:1 after incubation. One microliter aliquot of the reaction solution was then diluted 10-fold with RuBisCO reaction mix. The reaction mixture contained 50 mM HEPES–KOH (pH 7.8), 10 mM NaHCO_3_, 20 mM MgCl_2_, 0.2 mM NADH, 5 mM ATP, 5 mM phosphocreatine, 5 U creatine phosphokinase, 5 U glyceraldehyde-3-phosphate dehydrogenase (Sigma-Aldrich G2267), 5 U 3-phosphoglycerate kinase (Sigma-Aldrich P7634). The creatine phosphokinase was purified previously as a component of the customized PURE system (30). The absorbance of the reaction solution was then measured using a spectrophotometer (UV-2550, SHIMADZU) under two conditions: 0 min and 30 min incubated at 30°C. Using the absorbance data obtained, the background noise in the reaction solution was removed, and the integrated value of the specific peak at 340 nm was shown as A_340_.

### 2.3 SDS-PAGE analysis

**Figure 1.**
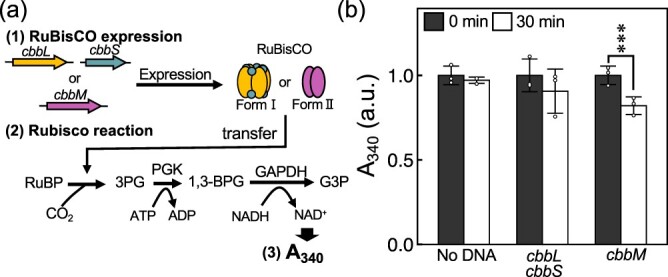
**NADH absorbance assay for two types of RuBisCO. (a)** Scheme of the assay method. In the first reaction, each RuBisCO was expressed in a PURE system (PUREfrex 2.0) at 30˚C for 4 h. An aliquot of each reaction solution was diluted with the Rubisco reaction mix, which contains RuBP, NaHCO_3_, 3-phosphoglycerate kinase (PGK), glyceraldehyde-3-phosphate dehydrogenase (GAPDH), ATP and NADH. The second reaction mixture was incubated at 30˚C for 30 min, during which RuBisCO fixes CO_2_ and converts RuBP to 3PG, oxidizing NADH to NAD^+^ in the process of synthesizing G3P in the downstream enzymatic reaction. The specific absorbance of NADH (A_340_), was measured at 0 and 30 min using a spectrophotometer. (b) A_340_ before and after reactions with and without each RuBisCO gene. Error bars represent the standard deviations of three independent experiments. The significance test was performed by Student’s *t*-test (***P  < 0.001).

SDS-PAGE to determine the translation amount of RuBisCO in Supplementary Figure S1 was performed as follows. First, the samples expressed in the experiment in Figure 1 were diluted in a stock buffer (50 mM HEPES–KOH (pH7.6), 100 mM KCl, 10 mM MgCl_2_, 7 mM 2-mercaptoethanol and 30% glycerol) at a ratio of 1/10 for *cbbL* and 1/3 for *cbbM*. Then, 1 µl of these samples were incubated in SDS sample buffer (50 mM Tris–HCl (pH 7.4), 2% SDS, 0.86 metre 2-mercaptoethanol and 10% glycerol) at 95°C for 5 min and subjected to 10% SDS-PAGE. The band intensities were analyzed using ImageJ, and the amount of translation was estimated from the calibration curve generated by bovine serum albumin (BSA), which was simultaneously subjected to SDS-PAGE shown in Supplementary Figure S1.

### 2.4 ATP production assay

Linear DNA encoding either *cbbM* or *cbbL* (5 nM) was incubated at 30°C for 14 h using the customized PURE system containing glycolytic enzymes. The *cbbL* was used as a control (mock) because CbbL, a large subunit of form I RuBisCO, is inactive without the small subunit and similar size as CbbM. The composition is shown in Supplementary Table S1 and contains phosphoglycerate mutase 1 (abcam, ab289791), enolase (Sigma-Aldrich, E6126) and pyruvate kinase (Roche, 10 128 155 001). The customized PURE system used here consists of highly purified protein components to avoid kinase contamination. Each component was purified through additional column purification in a stringent buffer as described previously (30). After incubation, 1 µl aliquots of the reaction mixture were diluted 50-fold with a luciferase-based ATP assay kit (Fujifilm-Wako, 340–09791), inverted four times, and immediately measured for oxyluciferin emission using a Luminometer (GloMax, Promega). Quantitation of synthesized ATP was calculated from a calibration curve using known concentrations of ATP.

### 2.5 Co-expression of RuBisCO and GFP

First, the linear DNAs encoding GFP (3 nM) and *cbbM* or *cbbL* (as a mock) (5 nM) were incubated for 16 h at 30°C using the customized PURE system including glycolytic enzymes. The composition is shown in Supplementary Table S2. GFP fluorescence was measured every 15 min until 4 h and every 30 min thereafter (Mx3005P, Agilent Technologies). The fluorescence value at 16 h was used as the GFP fluorescence after subtracting the background fluorescence value at 0 h.

### 2.6 Co-expression of RuBisCO and GFP in giant vesicles

The preparation of giant vesicles (GV) was based on the methods of a previous study by Shimane *et al.* (31) using1-palmitoyl-2-oleoyl-sn-glycero-3-phosphocholine (POPC, Avanti). The inner solution was the same as the reaction mixture for the experiment shown in Figure 3 except for additionally containing 200 mM sucrose. The composition of the outer solution is shown in Supplementary Table S2. After incubation at 30°C for 16 h, 2 µl of the reaction solution was subjected to confocal fluorescence microscopy (TCS SP8, Leica). GFP fluorescence values of GVs were analyzed by ImageJ.

## 3. Results

### 3.1 NADH absorbance assay for two forms of RuBisCO expressed in the PURE system

As candidates of RuBisCO genes expressed *in vitro*, we chose two RuBisCO genes from a cyanobacterium (*S. elongatus* PCC6301) and a photosynthetic bacterium (*R. rubrum*) because these genes have been expressed in their active form in *E. coli* in previous studies (32, 33). The *S. elongatus* RuBisCO (Form II) is a heterohexadecamer of two gene products (*cbbS* and *cbbL*), whereas the *R. rubrum* RuBisCO (Form II) is a homodimer of one gene product (*cbbM*).

The assay scheme is described in Figure 1a. First, DNA encoding the above RuBisCO genes (5 nM) was incubated in the commercial PURE system at 30°C for 4 h to express RuBisCO. Aliquots of the solution were then diluted with RuBisCO reaction solution and incubated at 30°C for 30 min. If the RuBisCO synthesized in the first reaction is sufficiently active, the synthesized RuBisCO catalyzes the conversion of CO_2_ and ribulose 1,5-bisphosphate (RuBP) to 3PG in the second reaction. Then, 3PG is further converted to glyceraldehyde 3-phosphate (G3P), 1,3-bisphosphoglycerate (1,3-BPG) and glyceraldehyde 3-phosphate (G3P) successively in the downstream enzymatic reactions. NADH is oxidized to NAD^+^ in the final step of this reaction pathway. We measured the NADH-specific absorbance (A_340_) before and after the reaction.

In the first reaction in the PURE system, CbbL (52 kDa) and CbbM (50 kDa) were expressed at ∼50 ng/µl (Supplementary Figure S1). We then measured A_340_ after the second reaction. We observed a significant decrease in A_340_ (i.e. decrease in NADH) with *cbbM* from *R. rubrum* compared to no DNA (i.e. negative control), but not with *cbbS and cbbL* from *S. elongatus* (Figure 1b). This result indicates that RuBisCO derived from *R. rubrum* was expressed in its active form in the PURE system.

### 3.2 ATP production by RuBisCO in the PURE system

**Figure 2.**
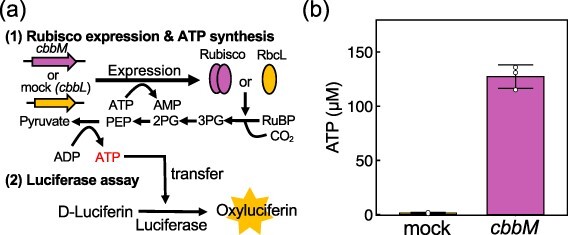
**ATP production by RuBisCO.** (a) Scheme of the assay method. In the first reaction, RuBisCO (*cbbM*) or mock (*cbbL*) was expressed in the customized PURE system containing glycolytic enzymes, RuBP, NaHCO_3_ and ADP, excluding ATP regeneration enzymes and creatine phosphate. The composition is shown in Supplementary Table S2. Through this reaction, RuBisCO and downstream glycolytic enzymes synthesize ATP through the process of converting RuBP to pyruvate. An aliquot of each reaction solution was used for the luciferase-based ATP assay. Oxyluciferin emission was measured using a luminometer. The synthesized ATP was quantified based on the calibration curve using known concentrations of ATP shown in Supplementary Figure S2. (b) ATP concentration after reactions with *cbbM* or mock. Error bars represent the standard deviations of three independent experiments. The raw data plot for oxyluciferin emission is shown in Supplementary Figure S2.

Next, we coupled the expressed RuBisCO activity to energy synthesis by introducing some reactions in glycolysis, which produces ATP through the process from 3PG, synthesized by RuBisCO, to pyruvate (25). The assay scheme is shown in Figure 2a. First, DNA encoding the *cbbM* or mock gene (*cbbL*) (5 nM) was incubated at 30°C for 14 h in the customized PURE system with glycolytic enzymes (phosphoglycerate mutase, enolase, pyruvate kinase), RuBP, NaHCO_3_ and ADP. For this PURE system, some enzymes for ATP regeneration (nucleoside diphosphate kinase and myokinase) and creatine phosphate were omitted, and creatine kinase was also reduced to 1/10 concentration. Residual ATP was then quantified by oxyluciferin luminescence. Since the ATP regeneration system (i.e. ATP regeneration from creatine phosphate by kinases) was omitted in the PURE system used here, ATP was consumed during the translation reaction and was almost undetectable without RuBisCO expression (Figure 2b, **mock**). In contrast, ∼120 µM ATP remained when CbbM RuBisCO was expressed (Figure 2b, ***cbbM***). This result suggests that ATP can be synthesized by the activity of CbbM RuBisCO. The residual ATP concentration was comparable (∼50%) to the normal PURE system that contains the standard ATP regeneration system (Supplementary Figure S2), which suggests that ATP production by expressed RuBisCO and glycolytic enzymes can be utilized as an ATP regeneration method in the PURE system.

### 3.3 Translational energy production by RuBisCO

**Figure 3.**
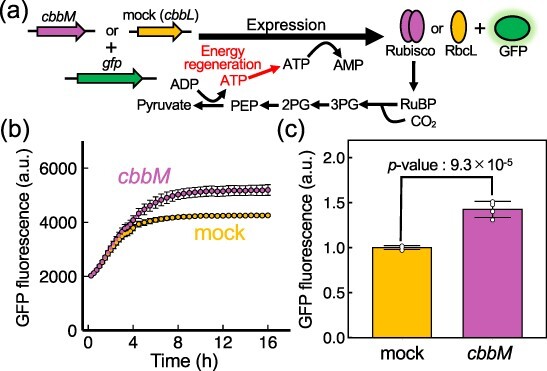
**ATP regeneration by RuBisCO for GFP translation.** (a) Schematic representation of the reaction. DNA encoding *cbbM* or mock genes and DNA encoding GFP were incubated in the custom PURE system, and GFP fluorescence was measured. Through this reaction, RuBisCO and downstream glycolytic enzymes synthesize ATP through the process of converting RuBP to pyruvate. The generated ATP can be used for the GFP translation reaction. (b) The time-course data of GFP fluorescence. Error bars represent the standard deviations of four independent experiments. (c) Relative GFP fluorescence at 16 h with cbbM or mock. Error bars represent the standard deviations of four independent experiments. The significance test was performed by Student’s *t*-test.

To test the possibility that the ATP production by expressed RuBisCO and glycolytic enzymes can be utilized as an ATP regeneration system in the PURE system, we incubated two DNA templates encoding *cbbM* (or mock gene) and *gfp* (5 nM and 3 nM, respectively) in the customized PURE system at 30°C for 16 h, and GFP fluorescence was measured (Figure 3a). We observed that the increase in GFP fluorescence with *cbbM* lasted up to about 8 h, whereas the increase with mock stopped at about 4 h (Figure 3b). The final amount of GFP fluorescence was also significantly higher with *cbbM* than with mock (Figure 3c). These results suggest that ATP production by expressed RuBisCO can be used as a source of translational energy for the PURE system.

### 3.4 Translational energy production by RuBisCO in giant vesicles

**Figure 4.**
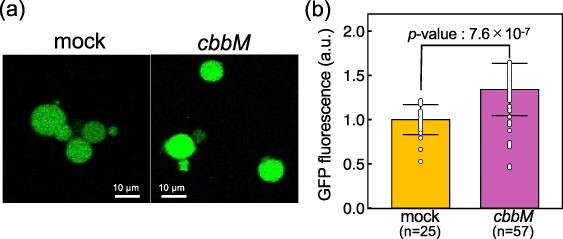
**Translational energy production by RuBisCO in GV. (a)** Confocal microscopy images of GFP expressed GVs. The scale bar indicated 10 µm. Differential interference contrast (DIC) and merged images with GFP fluorescence are shown in Supplementay Figure S3. (b) Expression of GFP with cbbM or mock. GFP fluorescence within GVs was calculated by ImageJ. The number of GVs that could be quantified is shown below the gene name. Error bars represent the standard deviations and the significance test was performed by Student’s *t*-test.

Compartmentalization of this system is necessary for the development of artificial cells that use RuBisCO to take up CO_2_ and also for the directed evolution of RuBisCO. Finally, we examined whether the RuBisCO-mediated ATP production system via CO_2_ fixation also works in giant vesicles (GVs). We incubated GVs prepared by the droplet transfer method (31) with the reaction system shown in Figure 3. The GVs containing *cbbM* expression showed ∼1.4 times higher GFP fluorescence than those containing mock gene expression (Figure 4), consistent with the result in the bulk reaction shown in Figure 3. These results demonstrate that the RuBisCO-mediated ATP production system is applicable to the artificial cell platform.

## 4. Discussion

Energy production within artificial cells is important for maintaining homeostatic artificial cell activity (19, 20). In this study, we synthesized RuBisCO, the enzyme that plays a central role in biological CO_2_ fixation, in a cell-free expression system and used its metabolism to synthesize ATP. First, we synthesized two forms of RuBisCO possessed by a cyanobacterium and a photosynthetic bacterium in a cell-free expression system and found that RuBisCO from *R.rubrum*, which is Form II, is active (Figure 1b). Next, we used this RuBisCO and a glycolytic enzyme to create a RuBisCO-dependent ATP production system (Figure 2b). Furthermore, ATP synthesized by this system was used as an energy source for translation reactions in the bulk and GV (Figures 3 and 4). Although there is room for improvement, these results represent a step toward the development of artificial cells capable of producing energy via CO_2_ fixation.

One limitation of the RuBisCO-based energy regeneration system is its low ATP regeneration ability. This system maintained only ∼3% of the initial ATP concentration after the reaction (Figure 2b). However, this limitation cannot be solely attributed to the low activity of RiBisCO because even the standard energy regeneration system of the PURE system that uses creatine phosphate and creatine phosphokinase can regenerate only ∼6% of the initial ATP concentration (Supplementary Figure S2). To maintain a stable ATP concentration in cell-free systems, it is imperative to incorporate a mechanism that allows continuous uptake of substrates by mimicking the behavior of living organisms.

One new aspect of this study is the successful synthesis and activity detection of RuBisCO in the PURE system. Previous biochemical studies on RuBisCO have been conducted through protein synthesis and purification through heterologous expression (34–37). However, these methods are laborious and time-consuming. The activity assay method developed in this study, which combines NADH absorbance assay and PURE system expression, does not require protein purification and can measure the activity of RuBisCO in a few hours. In addition, the PURE system consists of the minimum number of factors necessary for gene expression. Therefore, the concentration of proteins and substrates can be easily adjusted and the background noise caused by the contamination of other enzymes is minimized. These advantages make it possible to simultaneously examine various RuBisCO expressions and reaction conditions. The limitation of our results is that we could not detect the activity of *S. elongatus* RuBisCO, which could be resolved by the addition of chaperones or RuBisCO activase that contribute to the folding of RuBisCO, as in previous studies (34, 37, 38).

Another value of our results is the potential for directed evolution of RuBisCO under cell-free conditions. RuBisCO is the central enzyme for CO_2_ fixation, but its low catalytic activity due to its poor substrate selectivity has long been the subject of molecular evolutionary engineering (24, 39). Previous studies on the molecular evolution of RuBisCO have focused on photosynthetic selection and RuBisCO-dependent *E. coli* (32, 37). These evolutionary experiments have selected mutants that can improve solubility and kinetics (40–46). However, these approaches are limited by metabolic remodeling such that cell survival depends on RuBisCO activity (37, 46). Our results showed that ATP synthesis via CO_2_ fixation of expressed RuBisCO promoted GFP expression (Figures 2**–**4). This reaction has the same trend in the two environments, bulk and GV, and the activity of RuBisCO can be detected by measuring the final GFP fluorescence. This means that this system can be used for the in vitro evolution of RuBisCO using GFP fluorescence. For example, our system could be combined with microfluidic technology to select active RuBisCO genes (47–49). Furthermore, since this system is decoupled from cell survival, the CO_2_/O_2_ ratio and temperature can be freely regulated, allowing RuBisCO with various properties to be subjected to evolution in the future.

## Supplementary Material

ysad016_Supp

## Data Availability

Data is available on request from the authors.

## Material availability

Requests for materials should be addressed to N.I.
