# Supplementary material for: Cell-free expression of RuBisCO for ATP production in the synthetic cells
Source: Synth Biol (Oxf). 2023 Dec 20;8(1):ysad016. doi: 10.1093/synbio/ysad016 (PMC10750972; doi:10.1093/synbio/ysad016)
Supplement: ysad016_Supp [file ysad016_supp.zip › suppl_data/231027_Sugii_Supplement_6.pdf]

Supplementary Information for

Cell-Free Expression of RuBisCO for ATP Production in the Synthetic Cells.

Shugo Sugii, Katsumi Hagino, Ryo Mizuuchi, Norikazu Ichihashi

This file includes:

Figs. S1-3

Tables S1-S2

Supplemental Text

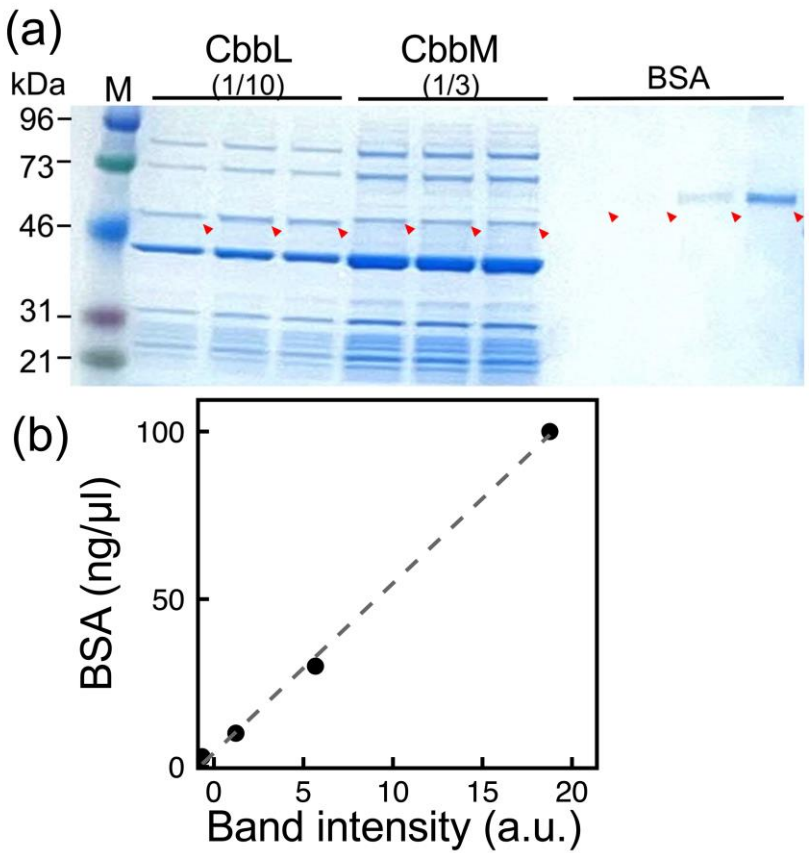

Figure S1 SDS-PAGE analysis of RuBisCO.

(a) Gel image of SDS-PAGE and CBB staining. Each of the three RuBisCO protein samples subjected is an independently expressed biological triplicate. The expected bands of each RuBisCO proteins (CbbL : 52 kDa, CbbM : 50 kDa) and BSA are indicated by the red arrowheads. CbbS was not identified due to the small size (13 kDa). (b) Calibration curve to estimate concentration of expressed RuBisCO. This curve was drawn using known concentrations of BSA.

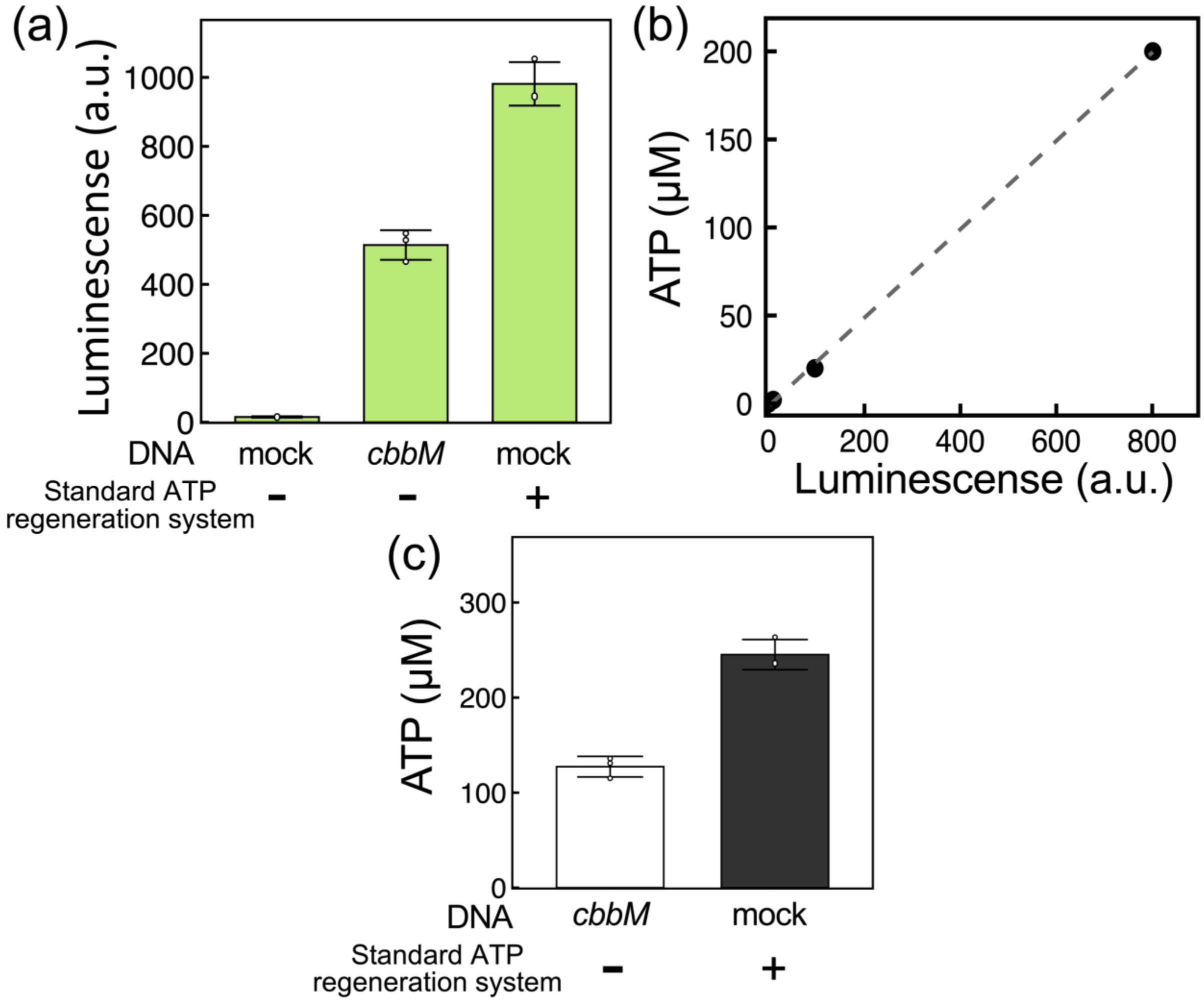

Figure S2 Raw data luminescence in Fig.2

(a) Raw data plots of luminescence in Fig. 2b. The labels at the bottom of the panel indicates the expressed DNA and whether the standard ATP regeneration system was included in the PURE system used here. Error bars represent the standard deviations of three independent experiments. (b) Calibration curve to estimate concentration of ATP. (c) Comparison of ATP synthesis ability between the normal PURE system that contains the standard ATP regeneration system (mock) and that lacks the ATP regeneration system but expresses a RuBisCO (cbbM).

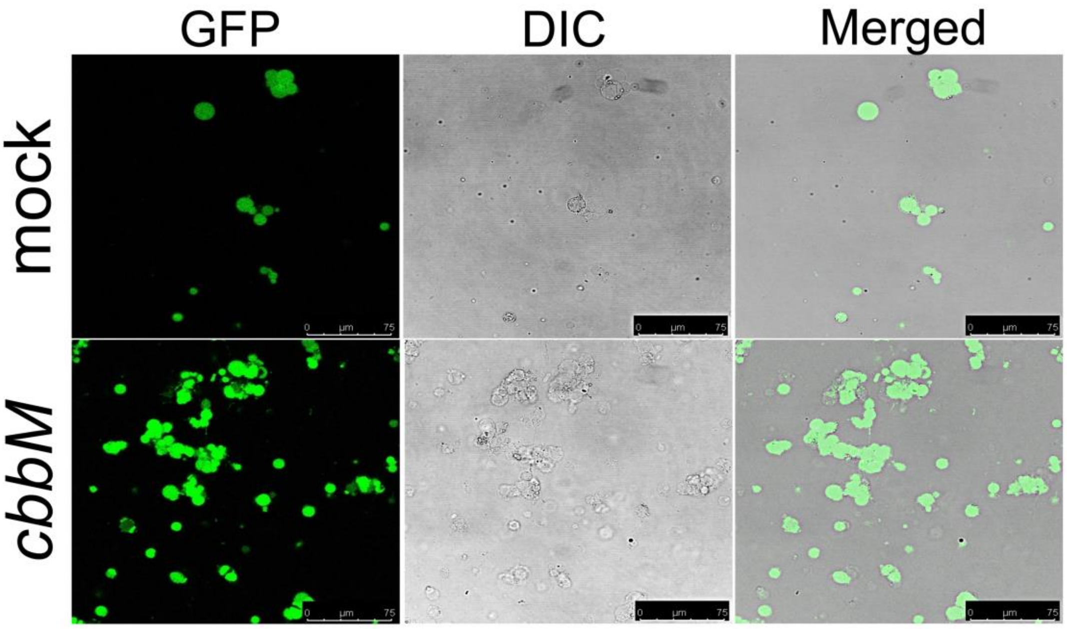

Figure S3 Confocal microscopy images of GFP expressed GVs.

GFP: Fluorescence image, DIC: differential interference contrast, Merged: Merged image GFP and DIC.

Table S1. Composition of the customized PURE system

Initiation factor 1	25 µM	ValRS	17 nM
Initiation factor 2	1 µM	Methionyl-tRNA formyltransferase	590 nM
Initiation factor 3	4.9 µM	Creatine kinase	25 nM
Elongation factor G	1.1 µM	Pyrophosphatase	41 nM
Elongation factor Tu	80 µM	Trigger factor	1 µM
Elongation factor Ts	3.3 µM	*E. coli* DEAH type RNA helicase A	100 nM
Release factor 1	49 nM	70S ribosome	1 µM
Release factor 2	48 nM	RNase inhibitor (Promega)	0.1 U/µL
Release factor 3	170 nM	T7 RNA polymerase (Takara)	0.42 U/µL
Ribosome recycling factor	3.9 nM	Tyrosine	0.3 mM
AlaRS	730 nM	Cysteine	0.3 mM
ArgRS	31 nM	18 other amino acids	0.36 mM
AsnRS	420 nM	tRNA mix (Roche, *E. coli*)	3.12 mg/mL
AspRS	120 nM	ATP	3.75 mM
CysRS	24 nM	GTP	2.5 mM
GlnRS	60 nM	CTP	1.25 mM
GluRS	230 nM	UTP	1.25 mM
GlyRS	86 nM	N-2-hydroxyethylpiperazine-N'-2-ethanesulfonic acid (pH 7.6)	100 mM
HisRS	85 nM	Glutamate acid potassium salt	280 mM
IleRS	370 nM	Spermidine	1.5 mM
LeuRS	41 nM	Magnesium acetate	18 mM
LysRS	120 nM	Dithiothreitol	1.5 mM
MetRS	110 nM	10-formyl-5,6,7,8-tetrahydro folic acid	10 µg/mL
PheRS	130 nM	Phosphoglycerate mutase 1	5 µg/mL
ProRS	170 nM	Enolase	1 µg/mL
SerRS	78 nM	pyruvate kinase	5 µg/mL
ThrRS	84 nM	ADP	2 mM
TrpRS	28 nM	Ribulose 1,5-bisphosphate	10 mM
TyrRS	150 nM	$NaHCO_3$	10 mM

Table S2. Composition of the outer solution

Component	Concentration
Tyrosine	0.3 mM
Cysteine	0.3 mM
18 other amino acids	0.36 mM
GTP	2.5 mM
CTP	1.25 mM
UTP	1.25 mM
N-2-hydroxyethylpiperazine-N'-2-ethanesulfonic acid (pH 7.6)	100 mM
Glutamate acid potassium salt	280 mM
Spermidine	1.5 mM
Magnesium acetate	18 mM
Dithiothreitol	1.5 mM
10-formyl-5,6,7,8-tetrahydro folic acid	10 µg/mL
ADP	2 mM
Ribulose 1,5-bisphosphate	10 mM
$NaHCO_3$	10 mM
Glucose	200 mM
